# Sequencing of a *Thermoanaerobacter kivui* isolate from DSMZ stock: major differences with reference assembly

**DOI:** 10.1128/mra.01250-24

**Published:** 2025-02-06

**Authors:** Rémi Hocq, Gerhard G. Thallinger, Stefan Pflügl

**Affiliations:** 1 Institute of Chemical, Environmental and Bioscience Engineering, Technische Universität Wien, Vienna, Austria; 2 Christian Doppler Laboratory for Optimized Expression of Carbohydrate-active Enzymes, Institute of Chemical, Environmental and Bioscience Engineering, TU Wien, Vienna, Austria; 3 Institute of Biomedical Informatics, Graz University of Technology, Graz, Austria; SUNY College of Environmental Science and Forestry, Syracuse, New York, New York, USA

**Keywords:** acetogens, thermophilic anaerobes, gas fermentation, wood-ljungdahl pathway, carbon dioxide fixation

## Abstract

*Thermoanaerobacter kivui* DSM 2030 is an industrially relevant microbe, whose genome has been available since 2014. As the stock obtained from DSMZ appeared to be multiclonal, we obtained a complete genome sequence by *de novo* assembly of an isolate from the DSMZ stock and reported differences to the initial assembly.

## ANNOUNCEMENT

*Thermoanaerobacter kivui* DSM 2030 is a thermophilic, Gram-positive, strictly anaerobic acetogenic bacterium (1), sparking interest due to its ability to ferment CO and CO_2_ into renewable fuels and chemicals (2). A fully assembled and annotated genome has been available since 2014 (NZ_CP009170.1, 3). Upon reception of the stock from DSMZ, we performed whole genome sequencing, which showed the culture was multiclonal, and therefore, we sequenced a random, well-isolated colony from this stock with a combination of long- (Oxford Nanopore Technology, ONT) and short-read (Illumina) technologies.

A *Thermoanaerobacter kivui* DSM 2030 stock was autotrophically grown in a mineral medium on H_2_ and CO_2_ (80:20) as sole carbon and energy sources (4, 5). Cells were subcultured twice and plated on a solid medium supplemented with glucose and yeast extract (4, 5). An isolated colony (named G-1) was sequenced.

Long-read sequencing: gDNA was extracted by treating cells with lysozyme (30 min, 10 mg mL^−1^ in 10 mM TE buffer) and by using the Zymo Quick DNA miniprep kit according to the manufacturer’s specifications. Extracted gDNA was sent to PlasmidSaurus (Eugene, OR, USA) for ONT sequencing. The library was prepared with the Rapid Barcoding Kit 96 V14 (SQK-RBK114.96, ONT) according to the manufacturer’s instructions, without DNA shearing or size selection. Sequencing was performed on a PromethION with an R10.4.1 flowcell. Basecalling, removal of low-quality reads, barcode splitting, and adapter trimming were performed with Guppy (v.6.4.6) in super-high accuracy mode, which resulted in 315,011 raw reads (*N*_50_ = 5,812). Down sampling to 100× coverage retaining the longest reads was performed with Seqkit v2.4.0 (6), resulting in 16,349 reads (*N*_50_ = 15,192).

Short-read sequencing: DNA extraction and sequencing were performed by Microsynth AG (Balgach, Switzerland). Cells were first treated with 15 mg mL^−1^ lysozyme (overnight, 37°C in Saline/Tris/EDTA/Triton* X-100 buffer). 20 µL proteinase K (20 mg mL^−1^), 20 µL RNase A (10 mg mL^−1^), and SDS (0.5% final) were added (10 min at RT, then 56°C for 2 hours). Cell debris was removed by centrifugation, and DNA was isolated with Qiagen DNeasy kit according to the manufacturer’s instructions. The sequencing library was constructed using Illumina’s DNA Prep tagmentation library preparation kit according to the manufacturer’s recommendations. The library was sequenced with an Illumina NovaSeq 6000 on an SP flow cell (paired-end, 250 bp reads). Paired-end reads (2,319,063) were quality filtered and trimmed with Trimmomatic v.0.39 (7).

Assembly was performed with Canu v2.2 (8), yielding a single linear 2,417,215 bp contig. The contig was polished with the trimmed Illumina reads using Minimap2 v2.24-r1122 (9) and HyPo v1.0.3 (10). The remaining conflicts were manually curated by visually inspecting the mapping in Integrative Genomics Viewer (11) and selecting the most likely bases; two ambiguous genomic loci were validated by PCR and Sanger sequencing (Supplementary Files).

Annotations were transferred from the published genome with Geneious Prime 2023.2.1 (https://www.geneious.com) and curated manually with a custom R (https://www.R-project.org) script. Parameter settings for all tools and R code are available on GitHub (https://github.com/ThallingerLab/ThermoanaerobacterKivui).

**Fig 1:**
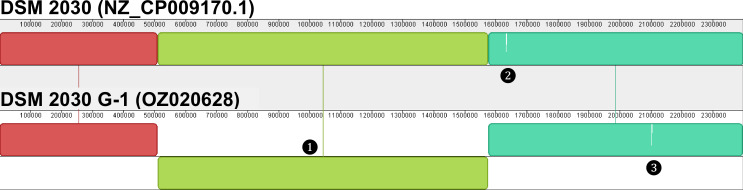
Sequence collinearity of the initial DSM 2030 genome with the newly assembled stock derivative (G-1) determined with mauve (13). Callouts mark the following: ❶ 1,063 kbps inversion including flanking 2,337 bps repeats, ❷ and ❸ translocation of a 1,602 bps ISLre2 family transposase gene from G-1 2,101,815–2,103,416 to DSM 2030 1,634,373–1,632,772. Colored lines connect collinear sequence segments across assemblies. White vertical bars within the chromosome represent sequences not present in the respective other assembly.

Overlaps of both ends of the linear contig were determined by BLAST v2.14.0 (12). After circularization (Geneious), polishing with Illumina reads, verification of four loci with Sanger sequencing, and rotation to *dnaA* as the start gene (Geneious), a 2,397,805 bp genome was obtained (35.0% GC, 308.5×/427.4× ONT/Illumina coverage). Annotation based on the published DSM 2030 genome yielded 2,516 predicted genes. G-1 and the published genome (3) differ by three structural variations and 11 SNVs/InDels (Fig. 1; Table 1).

**TABLE 1:** Sequence differences between the initial DSM 2030 genome (2,397,824 bps) and the sequenced G-1 clone (2,397,805 bps) obtained with NucDiff v2.0.3 (14) and manual curation^*a*^

Single nucleotide variations and InDels (≤ 50 bps)						
Start DSM 2030 (G-1)	End DSM 2030 (G-1)	Type (length)	Locus_tag DSM 2030 (G-1)	AA change	DSM 2030	G-1
110,469 (110,469)	110,469 (110,469)	Substitution (1)	TKV_RS00550 (TKVG1_00560)	*P*→A	C	G
542,777 (1,542,664)	542,798 (1,542,664)	Deletion (22)	TKV_RS02680 (TKVG1_08115)	Recovered frameshift	AGTAGGTAAGAGAGTAGCTGTA	–
704,424 (1,381,038)	704,424 (1,381,038)	Substitution (1)	TKV_RS03470 (TKVG1_07295)	T→S	T	A
704,605 (1,380,852)	704,605 (1,380,852)	Insertion (1)	Intergenic		–	T
880,963 (1,204,498)	880,963 (1,204,498)	Substitution (1)	TKV_RS04430 (TKVG1_06320)	V→L	G	C
1,062,261 (1,023,200)	1,062,261 (1,023,200)	Substitution (1)	TKV_RS05285 (TKVG1_05425)	–	A	G
1,559,965 (525,496)	1,559,965 (525,496)	Substitution (1)	TKV_RS07960 (TKVG1_02685)	N→D	T	C
1,976,392 (1,974,769)	1,976,392 (1,974,769)	Substitution (1)	TKV_RS10075 (TKVG1_10425)	G→R	C	G
2,117,471 (2,117,451)	2,117,471 (2,117,452)	Insertion (2)	Intergenic		–	TT
2,215,856 (2,215,836)	2,215,856	Deletion (1)	TKV_RS11295 (TKVG1_11685)	*P*→fs	A	–
2,338,172 (2,338,153)	2,338,172 (2,338,153)	Insertion (1)	Intergenic		–	A
2,342,672 (2,338,134)	2,342,672 (2,338,134)	Substitution (1)	TKV_RS11835 (TKVG1_12280)	–	T	A

Structural variations (>50 bps)						
Start	End	Type	Length (bps)	Comment		
508,843 (1,576,597)	1,576,618 (508,843)	Inversion	1,067,775	Inversion including flanking 2,337 bps repeats, which exhibit one SNV		
1,632,779	1,634,373	Translocation-deletion	1,602	Translocation to G-1 position 2,101,816–2,103,407 Restoration of TKV_RS08290 (TKVG1_08555) CDS, a sugar ABC permease		
2,103,437	2,103,437	Translocation-insertion	1,602	Translocation from DSM 2030 position 1,632,772–1,634,373		

^*a*^ Start and end position refer to the initial DSM 2030 assembly (NZ_CP009170.1, [3]).

## Data Availability

The *T. kivui* G-1 genome sequence has been deposited in ENA under accession OZ020628. The BioProject accession is PRJEB72631, and raw sequence reads are archived under accessions ERR13953883 (ONT) and ERR13432159 (Illumina).
